# Two Years of COVID-19 in Austria—Exploratory Longitudinal Study of Mental Health Outcomes and Coping Behaviors in the General Population

**DOI:** 10.3390/ijerph19138223

**Published:** 2022-07-05

**Authors:** Brigitte Lueger-Schuster, Irina Zrnić Novaković, Annett Lotzin

**Affiliations:** 1 Department of Clinical and Health Psychology, Faculty of Psychology, University of Vienna, 1010 Vienna, Austria; irina.zrnic@univie.ac.at; 2 Vienna Doctoral School in Cognition, Behavior and Neuroscience, University of Vienna, 1030 Vienna, Austria; 3 Department of Psychiatry and Psychotherapy, University Medical Center Hamburg-Eppendorf, 20246 Hamburg, Germany; a.lotzin@uke.de; 4 Department of Psychology, MSH Medical School Hamburg, 20457 Hamburg, Germany

**Keywords:** mental health, COVID-19, pandemic, coping, adjustment disorder, posttraumatic stress disorder, anxiety, depression, longitudinal study

## Abstract

Since the long-term mental health impact of COVID-19 is not yet fully understood, the present study explored changes in mental health outcomes and pandemic-related coping behaviors across four pandemic stages. The main objective was to gain insights into the dynamics of mental health and coping, considering different pandemic features at different assessment waves. The final sample consisted of *N* = 243 adults from the Austrian general population. Data were collected at four timepoints (between June 2020 and December 2021) via *LimeSurvey*, an open-source online survey tool. Symptoms of posttraumatic stress disorder (PTSD), adjustment disorder (AD), anxiety, and depression were assessed using validated instruments: Primary Care PTSD Screen for DSM-5 (PC-PTSD-5), AD-New Module 8 (ADNM-8), and Patient Health Questionnaire (PHQ4). We also administered the Pandemic Coping Scale (PCS) to address pandemic-related coping behaviors. Cochran’s Q test and repeated measures ANOVAs were applied to assess changes over time. The results indicated that prevalence rates of AD (χ^2^(2) = 16.88, *p* = 0.001), depression (χ^2^(3) = 18.69, *p* < 0.001), and anxiety (χ^2^(3) = 19.10, *p* < 0.001) significantly changed across four assessment waves. Changes in mean scores of the assessed mental health outcomes were also observed. For pandemic-related coping, we found differences in the subscales: healthy lifestyle: *F*(3, 651) = 5.11, prevention adherence: *F*(2.73, 592.35) = 21.88, and joyful activities: *F*(3, 651) = 5.03. Taken together, our study showed a higher mental health burden in wintertime than in summertime, indicating an increased need for psychosocial support in times of stricter measures, higher incidences, and higher death rates. Furthermore, the observed decrease in adaptive coping behaviors suggests that easy-to-implement coping strategies should be actively promoted in order to maintain mental health during and in the aftermath of pandemics.

## 1. Introduction

While Austria was not considered as one of the countries most severely affected by COVID-19 (i.e., infectious disease caused by the SARS-CoV-2 virus), it found itself in the spotlight of the pandemic early on following an outbreak in an Austrian ski resort, which contributed to the rapid spread of the virus in Europe [1]. Since March 2020, four hard nationwide lockdowns have been implemented, along with multiple strict regulations on the regional level, gravely impacting the daily lives of Austrian citizens (see Table 1). Specifically, in December 2021, Austria was the only country in Europe that was in a full lockdown. Given that preventive measures have been very strict not only at the beginning, but also at later pandemic stages, increased symptoms of mental health disorders could be expected.

During the first lockdown, increased levels of depression and anxiety symptoms were found in the Austrian general population [2]. A longitudinal study conducted from April to October 2020 revealed different patterns for different mental health outcomes: whereas suicidal ideation remained stable, and depressive and anxiety symptoms started increasing only shortly before the second lockdown, an immediate increase in domestic violence was observed following both lockdowns [3]. In Vienna, the capital of Austria, a significant increase in anxiety, depression, fatigue, and loneliness has been evident in the first pandemic year, with 46% of the participants representative of the Viennese population reporting mental health deterioration [4]. However, since prospective studies covering later stages of the pandemic are lacking, little is known about long-term trajectories of mental health in the Austrian population. For several other countries, longitudinal data on the mental health effects of the pandemic are more readily available, but with mostly short assessment periods. In the USA, Breslau et al. [5] applied a longitudinal design with two timepoints, showing higher psychological distress during the pandemic (May 2020) than before the pandemic (February 2019). In Spain, distinct patterns were present for symptoms of depression, anxiety, and posttraumatic stress disorder (PTSD): whereas anxiety and PTSD symptomatology showed a downward trend over three timepoints (at the beginning of the confinement, after a month, and after two months), depressive symptoms decreased only at the last assessment [6]. In the UK, a study conducted during the first 6 weeks of lockdown [7] noted increases in suicidal ideation, decreases in anxiety symptoms, and, remarkably, no significant changes in loneliness levels or depressive symptoms. In a German study covering a period of three months (May–June 2020) symptoms of mental health disorders were highest at the beginning, then remained stable or decreased only marginally, pointing toward the presence of a habituation mechanism evolving over time [8]. Similarly, a prospective English study observed a rapid decline in mental health problems during the first weeks of lockdown, with symptoms plateauing as lockdown measures were progressively eased [9].

While the loosening of preventive measures represents a plausible explanation for the observed changes in mental health over time in relation to COVID-19, other factors might also account for these changes. For instance, daily COVID-19 infection rates and decreased human mobility have been associated with significant increases in the prevalence of anxiety and depression [10]. A positive link has also been found between mental health problems and cumulative stress, resulting from co-occurring pandemic-related stressors [11]. On the other hand, becoming accustomed to the “COVID-19 way of life” might lead to an improvement in mental health as pandemic progresses. Previous research has shown that people have the capacity to successfully adapt and even flourish following large-scale stressful events [12,13]. Psychological adjustment is likely to be shaped by distinct individual characteristics, but also by the socio-political context in different pandemic stages [14,15]. This highlights the need to consider country-specific factors when investigating and interpreting the mental health effects of the pandemic.

In light of the multiple pandemic stages, each bearing different challenges, coping behaviors of the general population are likely to change and evolve. Being flexible, i.e., paying attention to changing situational demands and responding to them by using varying cognitive, emotional, and behavioral strategies may become essential for maintaining stable mental health [16,17]. An Italian longitudinal study conducted between March and May 2020 found lower levels of avoidant coping behaviors at baseline than at the final assessment [18]. Another Italian study launched only a month later noted an increased utilization of adaptive coping strategies at later timepoints [19]. Further longitudinal analyses of coping behaviors are urgently needed to better understand the mechanisms underlying psychological adjustments to COVID-19. Additionally, pandemic-related coping behaviors (e.g., adherence to containment measures; [20]) should be explored further.

Taken together, several international studies have investigated trajectories of mental health, but only a few have explored changes in coping behaviors. Most longitudinal studies have covered rather short intervals, e.g., several weeks [21] or a few months [8]. Studies that include almost the entire period of the pandemic up to this point and consider other mental health disorders are thus still lacking. Longitudinal analysis using data collected in several waves for a longer period would be particularly beneficial, considering the dynamics of the pandemic with regard to seasonal aspects, rates of infections, morbidity, and levels of restrictions, all of which may impact mental health and lead to potential habituation or (failed) adjustment mechanisms. For Austria, only a few studies on mental health during COVID-19 pandemic exist so far, and there are no studies on mental health and coping in later stages of the pandemic. Moreover, there are very little data on adjustment disorders (AD) in the Austrian general population.

The present study aims to address these research gaps by exploring the trajectories of various mental health outcomes and pandemic-related coping behaviors over 18 months of the pandemic in Austria. The main objective is to better understand the dynamics of mental health and coping, with specific aims to:

1. Determine the point prevalence of AD, PTSD, depression, and anxiety in the general population of Austria in four pandemic stages;
2. Compare the prevalence rates across the four stages;
3. Examine pandemic-related coping behaviors employed by the general population of Austria; and
4. Examine the differences in the use of coping behaviors across four pandemic stages.

## 2. Materials and Methods

The present study is part of an overarching project investigating the mental health impact of the COVID-19 pandemic across 11 European countries (ADJUST study; [22,23]). The study was preregistered in the OSF registry (https://doi.org/10.17605/OSF.IO/8XHYG). Each of the contributing countries collected data, with small modifications according to the local situation. Since Austria was the only country with a lockdown from November to December 2021, a fourth wave of data collection was administered in Austria, in addition to three waves conducted in all participating countries. The data reported in this paper stem from the general population sample of Austrian adults. The participants were assessed repeatedly at up to four assessment waves between June 2020 and December 2021 using open-source online survey tool *LimeSurvey*. Table 1 illustrates the COVID-19 situation in Austria during the four waves. Ethical approval was granted by the Ethics Committee of the University of Vienna, Reference Number: 00554. All participants provided informed consent prior to survey beginning.

### 2.1. Sampling

To increase the sample’s variability, participants of the first wave (baseline; T1) were recruited using different strategies. We distributed flyers in public spaces, shared our study link via social media, and contacted relevant stakeholders (e.g., universities, psychosocial support services), professional organizations (e.g., companies, theatres), as well as leisure and interest groups (e.g., gyms, clubs for senior citizens), asking them to distribute our study invitation to their staff or members. Interested individuals could access the survey by a website link or a QR code. After reading study information and providing consent on the first survey page, they could start answering our survey questions.

Inclusion criteria were: (1) age 18 or over, (2) sufficient skills in German language, and (3) willingness to participate. Participants who agreed to take part in the subsequent waves (*n* = 525) provided their e-mail and were re-contacted after approximately 6, 12, and 16 months (T2, T3 and T4, respectively).

### 2.2. Measures

The instruments relevant for the study are presented in the following section. The core set of instruments within the ADJUST study is described elsewhere [22].

The Adjustment Disorder New Module-8 (ADNM-8) is a brief measure of AD symptoms according to the ICD-11 [24]. It has demonstrated good reliability, convergent and factorial validity [24,25].

The Primary Care PTSD Screen for DSM-5 (PC-PTSD-5) is a 5-item screening measure for PTSD according to the DSM-5 criteria [26]. It has shown excellent diagnostic accuracy, high acceptability and good performance characteristics [27,28,29].

The 4-item Patient Health Questionnaire (PHQ-4) is an ultra-brief screening tool for depression and anxiety [30]. It consists of two subscales measuring depression (PHQ-2) and anxiety (GAD-2). Evidence for good psychometric properties and high applicability of PHQ-4 across different contexts has been provided [31].

The *Pandemic Coping Scale* (PCS) is a 13-item measure of coping behaviors specific for a pandemic [32]. The scale was constructed by a group of professionals in the field of traumatic stress, based on existing recommendations and studies on coping during a pandemic. It has shown sufficient factor validity and reliability [32].

The measures were assessed in all four waves. Internal consistency was determined using Cronbach’s α for all measures and all waves of assessment (for details see Table 2).

### 2.3. Data Analysis

Using the established cut-offs, we calculated prevalence rates of probable self-reported AD (ADNM-8 > 22), PTSD (PC-PTSD-5 > 3), depression (PHQ-2 > 2), and anxiety (GAD-2 > 2) for each timepoint. We then compared the differences in prevalence rates of AD, PTSD, depression, and anxiety between four timepoints using Cochran’s Q, followed by a post-hoc analysis. We then conducted one-way repeated measures ANOVAs to compare mean scores of all mental health outcomes and coping behaviors across four assessment waves. Finally, a two-way mixed ANOVA was used to test for gender differences in mental health outcomes and coping strategies.

As assessed by Normal Q-Q Plot, the variables mostly followed a normal distribution. The distributions of PHQ-4 and one PCS subscale were slightly left-skewed, which was not considered problematic, given the large sample size and application of analyses that are robust to violations of normality [33]. We applied the Bonferroni correction [34] to account for multiple tests in the post hoc analyses, and the Greenhouse-Geisser correction if the assumption of sphericity was violated (as assessed by the Mauchly’s test of sphericity).

Complete case analysis was used in order to avoid additional layer of measurement error in the data [35]. To rule out a possible bias introduced by the exclusion of incomplete cases, we performed multiple imputation using Fully Conditional Specifications [36,37] as sensitivity analysis. The analysis with estimated missing values in the mental health outcomes (*n* = 1001) revealed comparable results, as illustrated in the Appendix A.

Partial eta squared (η^2^) was applied as a measure of effect size, with η^2^ = 0.01, η^2^ = 0.06, and η^2^ = 0.14 reflecting a small, medium, and large effect, respectively [38]. All analyses were performed with SPSS Statistics Version 26 (IBM, Armonk, NY, USA).

## 3. Results

### 3.1. Sample Characteristics

After exclusion of participants not living in Austria at the time of the study or not completing any of the primary outcome measures, the original T1 sample included *N* = 1001 participants, of which 767 did not provide data on all relevant measures on all occasions. The final sample consisted of *N* = 234 participants, aged between 21 and 81 years (*M* = 48.75, *SD* = 15.03), responding to the four waves from all federal states of Austria. Their sociodemographic characteristics are shown in Table 3. Regarding health-related characteristics, the vast majority of participants described their health as “very good” (45.7%, *n* = 107) or “good” (32.9%, *n* = 77). One-fifth of the sample (19.7%, *n* = 46) indicated to be at risk for severe or life-threatening symptoms of the coronavirus disease, with old age and pre-existing conditions being the most frequently mentioned reasons for being at risk.

An independent-samples *t*-test was run to determine if there were differences in mean age and education between participants who responded to all waves and those who did not. Inspection of boxplots indicated there were no outliers in the data. Normal Q-Q Plot showed a normally distributed age in both samples. There was homogeneity of variances, as assessed by Levene’s test (*p* = 0.102 for age; *p* = 0.119 for education). The respondents were statistically significantly older (*M* = 48.75, *SD* = 15.03) than non-respondents (*M* = 44.59, *SD* = 13.83), *t*(999) = 3.95, *p* < 0.001. The respondents were also slightly better educated (*M* = 3.49, *SD* = 0.74) than non-responders (*M* = 3.30, *SD* = 0.80), *t*(999) = 3.32, *p* = 0.001. Respondents and non-respondents did not differ in terms of gender, as assessed by Fisher’s exact test, *p* = 0.771.

### 3.2. Longitudinal Changes in Mental Health Outcomes

The estimated prevalence rates of the assessed mental health outcomes at four timepoints are depicted in Table 4. The prevalence rates of self-reported probable AD differed significantly across four waves, χ^2^(2) = 16.88, *p* = 0.001 (*n* = 221). Pairwise comparison using Dunn’s test with a Bonferroni correction indicated that the prevalence rates statistically significantly changed between T1 and T2 (*p* = 0.001), and between T2 and T3 (*p* = 0.009). Regarding probable depression and anxiety disorder, the percentage of participants at risk was also statistically significantly different at the different time points, with χ^2^(3) = 18.69 for depression and χ^2^(3) = 19.10 for anxiety disorder (*p* < 0.001; *n* = 220). According to the Dunn’s test, statistically significant increase in the percentage of participants at risk for probable depression was detected between T1 and T4 (*p* = 0.02), and T3 and T4 (*p* = 0.001), whereas a significant decrease was detected between T2 and T3 (*p* = 0.02). Similarly, prevalence of probable anxiety disorder significantly increased between T1 and T4 (*p* = 0.01), and T3 and T4 (*p* < 0.001). The observed decrease between T2 and T3 was nearly significant, with *p* = 0.055.

After establishing the prevalence rates, we explored changes in mean scores of the assessed mental health outcomes. The AD, depression, and anxiety scores significantly changed across four assessment waves, with highest values at T2 and T4. Table 5 provides the results on the main effect of time, including post hoc analyses.

As shown in Table 6, the interaction effect between gender and time (GxT) on the assessed mental health outcomes was not statistically significant. Therefore, an analysis of the main effect for gender was performed, indicating significantly higher scores for female participants than male participants on all mental health outcomes across all timepoints, with the largest effect observed for anxiety symptoms. On average, females’ scores were 1.911 points (95% CI [0.45, 3.38]) higher for ADNM-8, 0.36 points (95% CI [0.03, 0.69]) higher for PHQ-2, and 0.62 points (95% CI [0.27, 0.98]) higher for GAD-2. All details regarding gender differences are depicted in Table 6.

Due to an extremely low number of participants who completed PC-PTSD-5 at all timepoints (*n* = 13), ANOVA could not be conducted for this measure. Thus, only mean values of PC-PTSD-5 at each timepoint are reported.

### 3.3. Longitudinal Changes in Pandemic-Related Coping Behaviors

One-way repeated measures ANOVA showed a significant decrease in the total score of PCS over time, *F*(2.89, 626.12) = 6.54, *p* < 0.001, partial η^2^ = 0.03. The highest mean score was observed at T1 (27.27 ± 6.33) and the lowest at T4 (25.86 ± 6.30), with T2 and T3 means in between (26.36 ± 6.44 and 26.34 ± 6.49). Changes on the PCS subscales between the four timepoints are presented in Table 7. As shown in Table 8, the interaction effect between gender and time (GxT) on the assessed coping behaviors was not statistically significant. Therefore, we conducted an analysis of the main effect for gender. Significantly higher scores for female participants were found on subscales “Healthy lifestyle” and “Daily structure”.

## 4. Discussion

The present study focused on adjustment disorder (AD) acknowledging that the dynamics of the pandemic has challenged the population’s adjustment capacity. Overall, we did not assess the pandemic as a traumatic experience because it is not life-threatening for the majority of the population. However, for some people, the pandemic includes life-threatening and traumatic aspects which our study also addressed. Moreover, we included depression and anxiety symptoms as common reactions to chronic stress. Thus, our longitudinal design could foster the planning of mental health services especially for the part of population that suffers from failed adjustment or a limited capacity to adjust to the pandemic. For this planning, the changes in coping over time in response to environmental factors, such as the stringency of preventive measures, might also be of relevance.

Specifically, this study compared pandemic-related coping behaviors and symptoms of AD, PTSD, depression, and anxiety during the 18 months of COVID-19 in Austria. Across the four assessment waves, we found rather high point prevalence rates and mean scores for all mental health outcomes. This held true for the complete-case analysis as well as the analysis using imputed missing values. The highest values for anxiety and depression were identified at T4 (December 2021), when Austria was under strict lockdown conditions. For all disorders we screened for, we observed lower prevalence and mean scores in summertime (T1 and T3), than in wintertime (T2 and T4), the latter being characterized by stricter regulations, higher incidence and death rates. In wintertime, the participants did not engage in as many joyful activities, indicating that pandemic fatigue or resignation in periods with stricter regulations might hinder adaptive coping behaviors. Furthermore, a gradual decrease in healthy lifestyle and prevention adherence suggests that even well-educated, older individuals did not fully comply with the pandemic regime for a longer period of time. This contradicts studies reporting more adaptive coping behaviors in later assessment waves [18], possibly due to the different timepoints implemented in our study. Maintaining daily structure, which had a positive effect on mental health in earlier COVID-19 studies [39] seemed to be equally relevant at all four timepoints.

With regards to gender differences, we observed higher values in women on all mental health outcomes at all timepoints. This finding is in line with the existing literature [10,23]. Although the trajectories of mental health slightly differed between male and female participants (e.g., depression score being highest at T2 in women, and at T4 in men), they followed the same pattern as in the overall sample, with fewer symptoms of mental health disorders reported in summertime than in wintertime. Regarding coping, women were more likely to use all pandemic-related coping behaviors at all timepoints. These gender differences have been observed in previous studies, with different coping behaviors being more common among female participants [17,40]. Further work is required to examine whether and how these differences in coping behaviors are related to mental health.

Overall, our results suggest that changes in the general population during COVID-19 pandemic depend on multiple factors. Both changes in mental health and in coping behaviors might be attributed to the changing pandemic situation, e.g., different incidence and containment measures, but might also depend on the season. Thus, our findings add to the studies covering shorter assessment intervals (e.g., [8]), by indicating that mental health and coping patterns change even in the later stages of the pandemic. Over time, individuals tend to adopt less healthy coping behaviors and manifest greater psychopathological symptoms. In order to provide adequate psychosocial support, the varying mental health challenges and needs of the general population need to be acknowledged.

Taken together, our study shows that the pandemic-related restrictions have left their mark on the people of Austria. Hence, it cannot be expected that everyone can successfully adjust to the changes caused by COVID-19. This highlights the need for a broad mental health campaign in order to address mental health needs and foster psychological adjustment in the general population, e.g., focusing on healthy behaviors and adaptive coping in periods of more restrictive measures. In addition, large mental health campaigns might deliver psychoeducation or inform the population how, where, and when to seek help. Finally, our findings on different mental health problems and coping behaviors might help to optimize psychosocial services all over Austria.

### Strengths and Limitations

To the best of our knowledge, this is the first longitudinal study on the mental health impact of COVID-19 covering almost all pandemic stages so far, with rather large intervals between the assessment waves. Apart from previously investigated trajectories of depression, anxiety, and PTSD (e.g., [3,6]), we also assessed changes in symptoms of AD. This is particularly relevant given that AD might be the most important, widespread mental health outcome of the pandemic [41]. It develops in response to stressors such as illness, relationship problems, financial or work-related issues, which are all relevant in this context [23,42]. Moreover, we provided some initial reports of pandemic-related coping behaviors adopted by the general population in different pandemic stages. Considering the long assessment period, another strength of the study is the relatively high number of participants who responded to all waves (45% of those who agreed to be re-contacted). Furthermore, the sample was well-distributed in terms of income and age.

An important limitation was that the participants were highly educated and predominantly female, which hampers the generalizability of the results. Although we have covered four distinct pandemic stages, we did not collect data in the very first months of the pandemic, which was declared to have begun in March 2020 [43]. Furthermore, while the internal consistency was satisfactory for most measures, low and unstable values were observed for some of the PCS subscales. Given the early stage, exploratory character of the study, we neither formulated hypotheses nor found it feasible to analyze possible predictors and groups at-risk. Associations between trajectories of mental health outcomes, coping behaviors, and sociodemographic characteristics other than gender should also be examined in future studies. Finally, our findings could not be contrasted with mental health trajectories before the pandemic, as, to our knowledge, no comparable epidemiological studies which included AD existed in Austria.

## 5. Conclusions

The present study paves the way for future research to explore the longitudinal impact of COVID-19 on mental health. Our findings highlight the need to consider governmental restrictions and the differing pandemic-related situations when investigating mental health trajectories. Importantly, findings of the present study also underline the importance of promoting easy-to-implement coping strategies (e.g., joyful activities) to foster healthy peri- and post-adjustment. Even in times of improving pandemic situation, the psychological burden of COVID-19 should be acknowledged and adequately addressed.

## Figures and Tables

**Table 1:** COVID-19 situation at four timepoints of data collection.

	T1	T2	T3	T4 ^a^
**Recruitment period**	27 June 2020– 22 September 2020	14 January 2021– 29 March 2021	13 July 2021– 08 October 2021	26 November 2021– 13 December 2021
**Duration of data collection**	88 days ≈ 13 weeks	75 days ≈ 11 weeks	88 days ≈ 13 weeks	18 days ^a^ ≈ 3 weeks
**Stringency index**				
First week of assessment	50	82.41	49.07	64.81
Last week of assessment	37.04	73.15	51.85	64.81
**Stringency index mean**	38.59	77.58	53.63	67.13
**Stringency index median**	37.96	75.93	51.85	67.59
**Incidence**				
First week of assessment	4.06/1 M	229.54/1 M	17.71/1 M	1482.74/1 M
Last week of assessment	78.95/1 M	362.96/1 M	200.80/1 M	459.72/1 M
**Deaths**				
First week of assessment	0.19/1 M	5.58/1 M	0.10/1 M	5.26/1 M
Last week of assessment	0.22/1 M	2.94/1 M	1.11/1 M	5.91/1 M
**Case fatality rate**				
First week of assessment	3.98%	1.78%	1.65%	1.10%
Last week of assessment	1.96%	1.72%	1.46%	1.07%

Note: All data are based on COVID-19 dataset provided by *Our World in Data* (https://github.com/owid/covid-19-data/tree/master/public/data, accessed on 9 May 2022). Stringency index: a composite measure of nine response metrics that records the strictness of government measures (from 0 to 100; i.e., 100 = strictest response). Case fatality rate (CFR): ratio between confirmed deaths and confirmed cases. M = million. 7-day rolling average was used for incidence (i.e., confirmed cases) and deaths. Cumulative values were used for the CFR. ^a^ The shorter period of data collection (compared to T1–T3) was reflecting the lockdown period, since the aim was to assess mental health during the lockdown. Furthermore, the then new Omicron-variant was already ante portas.

**Table 2:** Internal consistency of the included measures across four assessment waves.

	T1	T2	T3	T4
	Cronbach’s α (*n*)	Cronbach’s α (*n*)	Cronbach’s α (*n*)	Cronbach’s α (*n*)
**ADNM-8 ^a^**	0.92 (902)	0.92 (374)	0.93 (341)	0.92 (258)
Preoccupation	0.89 ^b^	0.89	0.91	0.90
Failure to adapt	0.85	0.84	0.85	0.84
**PHQ-4 ^a^**	0.83 (809)	0.86 (373)	0.89 (339)	0.88 (256)
Depression	0.75	0.81	0.83	0.83
Anxiety	0.76	0.80	0.84	0.84
**PCS ^a^**	0.82 (827)	0.81 (370)	0.83 (335)	0.81 (255)
Healthy lifestyle	0.80	0.78	0.81	0.78
Daily structure	0.86	0.83	0.86	0.89
Prevention adherence	0.45	0.47	0.53	0.33
Joyful activities	0.65	0.62	0.67	0.63
**PC-PTSD**	0.79 (866)	0.65 (59)	0.71 (64)	0.83 (54)

Note: ADNM-8 = Adjustment Disorder New Module-8; PHQ-4 = 4-item Patient Health Questionnaire; PC-PTSD = Primary Care PTSD Screen for DSM-5; PCS = Pandemic Coping Scale. The PHQ-4 has two subscales: depression (PHQ-2) and anxiety (GAD-2) subscale. The PCS has four subscales: healthy lifestyle, daily structure, prevention adherence and joyful activities. ^a^ Since the same number of participants was included in the calculation of Cronbach’s α for the total scale and its subscales, *n* is indicated only once per timepoint. ^b^ *n* = 903.

**Table 3:** Sociodemographic characteristics of the final sample (*N* = 234).

Characteristic	*n*	%
**Gender**		
Male	75	32.1
Female	158	67.5
Other	1	0.4
**Education**		
<10 years of schooling	1	0.4
≥10 years of schooling	31	13.2
Vocational studies	54	23.1
University degree	148	63.2
**Income ^a^**		
Very low	22	10.4
Low	70	33.0
Medium	20	9.4
High	100	47.2
**Work area**		
Health care	60	25.6
Public security	2	0.9
Retail/services	6	2.6
Maintenance/repair/construction	2	0.9
Education (e.g., teacher, lecturer)	28	12.0
Other	92	39.3
Not working	44	18.8
**Community**		
Large city	160	68.4
Suburb near a large city	16	6.8
Small city or town	39	16.7
Rural area	19	8.1
**Relationship status**		
Single	58	24.8
Temporary relationship(s)	6	2.6
Stable relationship, living separately	30	12.8
Stable relationship, living together	140	59.8
**Children**		
Yes	139	59.4
No	95	40.6

Note: ^a^ *n* = 212.

**Table 4:** Prevalence rates of probable mental health disorders at four timepoints.

	T1		T2		T3		T4	
	*n*	%	*n*	%	*n*	%	*n*	%
**Adjustment disorder** (ADNM-8) ^a^	29	13.1	50	22.6	32	14.5	41	18.6
**Posttraumatic stress disorder** (PC-PTSD-5) ^a^	17	7.7	1	0.5	5	2.3	10	4.5
**Depressive and anxiety disorders** (PHQ-4) ^b^:								
**Depression subscale (PHQ-2)**	30	13.6	44	20.0	24	10.9	50	22.7
**Anxiety subscale (GAD-2)**	28	12.7	38	17.3	23	10.5	46	20.9

Note: ADNM-8 = Adjustment Disorder New Module-8; PC-PTSD = Primary Care PTSD Screen for DSM-5; PHQ-4 = 4-item Patient Health Questionnaire; PHQ-2 = Depression subscale of PHQ-4; GAD-2 = Anxiety subscale of PHQ. The calculation of prevalence rates was based on the established cut-off scores: ADNM-8 > 22, PC-PTSD-5 > 3, PHQ-2 > 2 and GAD-2 > 2. ^a^ *n* = 221. ^b^ *n* = 220.

**Table 5:** Means, standard deviations, and repeated measures ANOVA statistics for mental health outcomes.

	T1	T2	T3	T4	ANOVA		Post Hoc Analysis	
	*M* (*SD*)	*M* (*SD*)	*M* (*SD*)	*M* (*SD*)	*F* Ratio	Partial η^2^	Group Comparison	Mean Difference
**Adjustment disorder (ADNM-8) ^a^**	14.86 (5.68)	16.65 (6.41)	15.13 (6.12)	16.31 (6.33)	*F*(2.86, 628.33) = 11.99 ***	0.05	T1 vs. T2	1.79 ***
							T1 vs. T3	0.27
							T1 vs. T4	1.45 **
							T2 vs. T3	−1.53 ***
							T2 vs. T4	−0.34
							T3 vs. T4	1.18 **
**Depression subscale (PHQ-2) ^b^**	1.29 (1.39)	1.66 (1.46)	1.16 (1.36)	1.66 (1.56)	*F*(3, 657) = 14.62 ***	0.06	T1 vs. T2	0.38 **
							T1 vs. T3	−0.13
							T1 vs. T4	0.37 **
							T2 vs. T3	−0.51 ***
							T2 vs. T4	0.01
							T3 vs. T4	0.50 ***
**Anxiety subscale (GAD-2) ^b^**	1.13 (1.39)	1.43 (1.57)	1.10 (1.38)	1.47 (1.61)	*F*(2.86, 625.50) = 10.23 ***	0.05	T1 vs. T2	0.30 **
							T1 vs. T3	0.04
							T1 vs. T4	0.34 **
							T2 vs. T3	−0.34 ***
							T2 vs. T4	0.04
							T3 vs. T4	0.34 ***
**Posttraumatic stress disorder (PC-PTSD-5)**	0.94 (1.37) ^a^	1.37 (1.24) ^c^	1.30 (1.47) ^d^	1.57 (1.81) ^d^				

Note: As adjustment for multiple comparisons, post hoc tests were performed with a Bonferroni correction. ^a^ *n* = 221. ^b^ *n* = 220. ^c^ *n* = 35. ^d^ *n* = 46. ** *p* < 0.01. *** *p* < 0.001.

**Table 6:** Descriptive statistics and two-way ANOVA statistics of mental health outcomes for males and females.

	Male		Female		ANOVA		
	*M*	*SD*	*M*	*SD*	Effect	*F* Ratio	Partial η^2^
**ADNM-8 ^a^**							
T1	13.14	4.54	15.63	5.99	GxT	*F*(2.86, 623.36) = 0.63	0.003
T2	15.27	6.33	17.25	6.37			
T3	14.09	5.36	15.57	6.41	G	*F*(1, 218) = 6.6 *	0.029
T4	15.13	5.74	16.82	6.54			
**PHQ-2 ^b^**							
T1	1.09	1.17	1.36	1.46	GxT	*F*(3, 651) = 1.39	0.006
T2	1.35	1.15	1.81	1.57			
T3	0.78	0.97	1.33	1.49	G	*F*(1, 217) = 4.74 *	0.021
T4	1.54	1.54	1.71	1.58			
**GAD-2 ^b^**							
T1	0.81	1.05	1.28	1.51	GxT	*F*(2.85, 618.72) = 0.92	0.004
T2	1.03	1.03	1.61	1.75			
T3	0.57	0.81	1.33	1.51	G	*F*(1, 217) = 1.88 ***	0.052
T4	1.01	1.31	1.68	1.69			
**PC-PTSD-5**							
T1 ^a^	0.53	0.88	1.12	1.52			
T2 ^c^	1.09	1.14	1.50	1.29			
T3 ^d^	1.06	1.00	1.43	1.68			
T4 ^e^	1.29	1.64	1.65	1.91			

Note: ADNM-8 = Adjustment Disorder New Module-8; PC-PTSD = Primary Care PTSD Screen for DSM-5; PHQ-4 = 4-item Patient Health Questionnaire; PHQ-2 = Depression subscale of PHQ-4; GAD-2 = Anxiety subscale of PHQ. The main effects of time for all mental health outcomes are presented in Table 5. ^a^ *n* = 220. ^b^ *n* = 219. ^c^ *n* = 35. ^d^ *n* = 46. ^e^ *n* = 45. * *p* < 0.05. *** *p* < 0.001.

**Table 7:** Means, standard deviations, and repeated measures ANOVA statistics for pandemic-related coping behaviors.

	T1	T2	T3	T4	ANOVA		Post Hoc Analysis	
	*M* (*SD*)	*M* (*SD*)	*M* (*SD*)	*M* (*SD*)	*F* Ratio	Partial η^2^	Group Comparison	Mean Difference
**Healthy lifestyle**	9.13 (3.59)	8.84 (3.58)	8.57 (3.73)	8.50 (3.38)	*F*(3, 651) = 5.11 **	0.02	T1 vs. T2	−0.29
							T1 vs. T3	−0.56 *
							T1 vs. T4	−0.63 **
							T2 vs. T3	−0.27
							T2 vs. T4	−0.34
							T3 vs. T4	−0.07
**Daily structure**	4.48 (1.69)	4.51 (1.56)	4.43 (1.74)	4.48 (1.69)	*F*(2.85, 617.29) = 0.16	0.00	*not conducted since ANOVA was not significant*	
**Prevention adherence**	5.61 (0.72)	5.48 (0.88)	5.39 (1.01)	5.15 (1.11)	*F*(2.73, 592.35) = 21.88 ***	0.09	T1 vs. T2	−0.12
							T1 vs. T3	−0.22 **
							T1 vs. T4	−0.50 ***
							T2 vs. T3	−0.10
							T2 vs. T4	−0.34 ***
							T3 vs. T4	−0.24 **
**Joyful activities**	8.06 (2.50)	7.53 (2.51)	7.95 (2.45)	7.74 (2.44)	*F*(3, 651) = 5.03 **	0.02	T1 vs. T2	−0.52 **
							T1 vs. T3	−0.10
							T1 vs. T4	−0.32
							T2 vs. T3	0.42 *
							T2 vs. T4	0.21
							T3 vs. T4	−0.22

Note: *n* = 218. As adjustment for multiple comparisons, post hoc tests were performed with a Bonferroni correction. * *p* < 0.05. ** *p* < 0.01. *** *p* < 0.001.

**Table 8:** Descriptive statistics and two-way ANOVA statistics of pandemic-related coping for males and females.

	Male		Female		ANOVA		
	*M*	*SD*	*M*	*SD*	Effect	*F* Ratio	Partial η^2^
**Healthy lifestyle**							
T1	8.54	3.66	9.40	3.55	GxT	*F*(3, 645) = 1.00	0.01
T2	7.88	3.87	9.28	3.37			
T3	7.68	3.73	8.97	3.67	G	*F*(1, 215) = 5.83 *	0.03
T4	7.90	3.58	8.79	3.27			
**Daily structure**							
T1	4.23	1.78	4.62	1.62	GxT	*F*(2.85, 611.90) = 2.52	0.01
T2	3.96	1.87	4.76	1.33			
T3	3.77	1.90	4.76	1.56	G	*F*(1, 215) = 15.42 ***	0.07
T4	3.91	1.82	4.74	1.57			
**Prevention adherence**							
T1	5.57	0.70	5.63	0.73	GxT	*F*(2.74, 587.98) = 0.25	0.00
T2	5.41	0.88	5.52	0.88			
T3	5.28	1.04	5.45	0.99	G	*F*(1, 215) = 0.99	0.01
T4	5.07	1.10	5.18	1.11			
**Joyful activities**							
T1	8.04	2.60	8.07	2.46	GxT	*F*(3, 645) = 1.16	0.01
T2	7.22	2.81	7.68	2.36			
T3	7.58	2.70	8.12	2.32	G	*F*(1, 215) = 1.62	0.01
T4	7.38	2.61	7.91	2.36			

Note: *n* = 217. Data from one participant identifying as “other” were not included in the analysis, given the very small subsample size (*n* = 1). * *p* < 0.05. *** *p* < 0.001.

**Figure A1:**
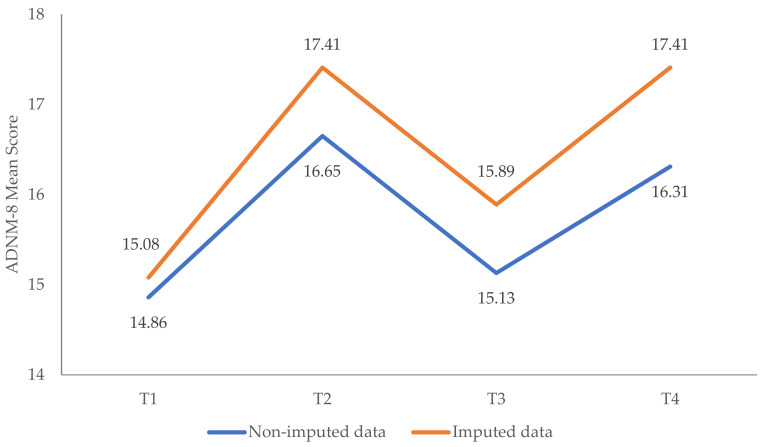
Changes in symptoms of adjustment disorder (ADNM-8) over time: imputed vs. non-imputed results. Note: Imputed dataset: *n* = 1001. Non-imputed dataset: *n* = 221.

**Figure A2:**
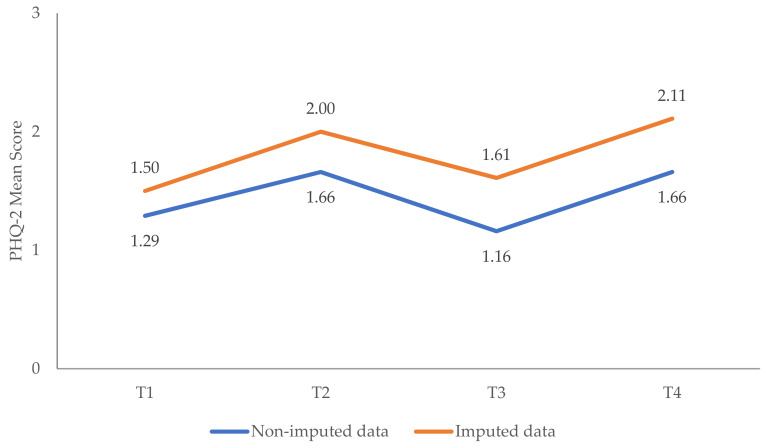
Changes in symptoms of depression (PHQ-2) over time: imputed vs. non-imputed results. Note: Imputed dataset: *n* = 1001. Non-imputed dataset: *n* = 220.

**Figure A3:**
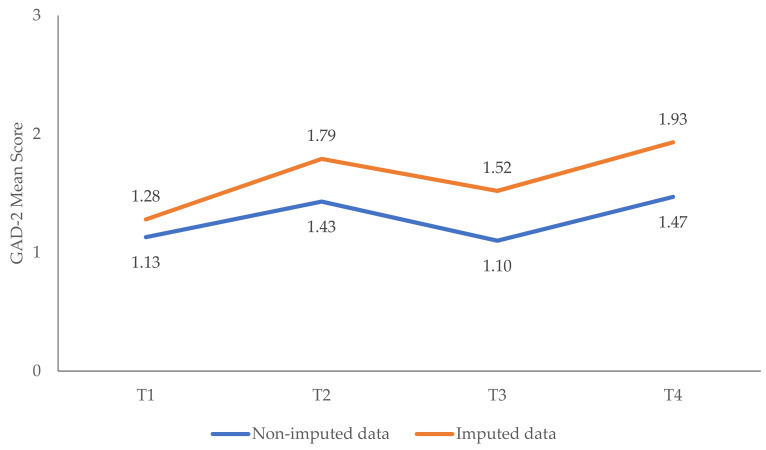
Changes in symptoms of anxiety (GAD-2) over time: imputed vs. non-imputed results. Note: Imputed dataset: *n* = 1001. Non-imputed dataset: *n* = 220.

**Figure A4:**
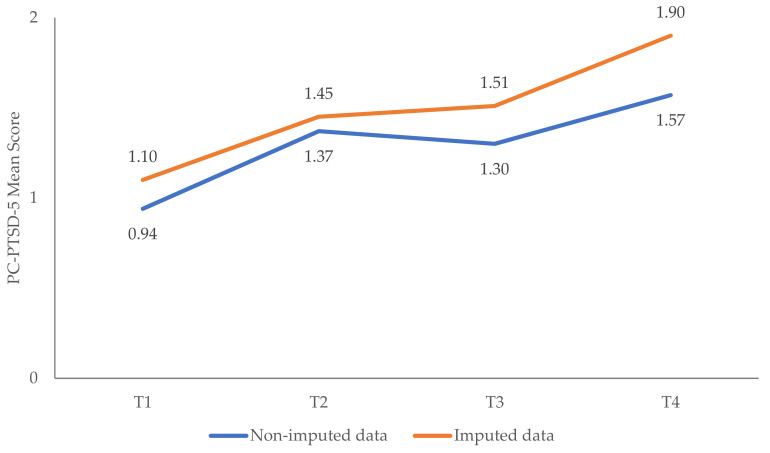
Changes in symptoms of PTSD (PC-PTSD-5) over time: imputed vs. non-imputed results. Note: Imputed dataset: *n* = 1001. Non-imputed dataset: *n*_t1_ = 221. *n*_t__2,3_ = 35. *n*_t__4_ = 46.

**Figure A5:**
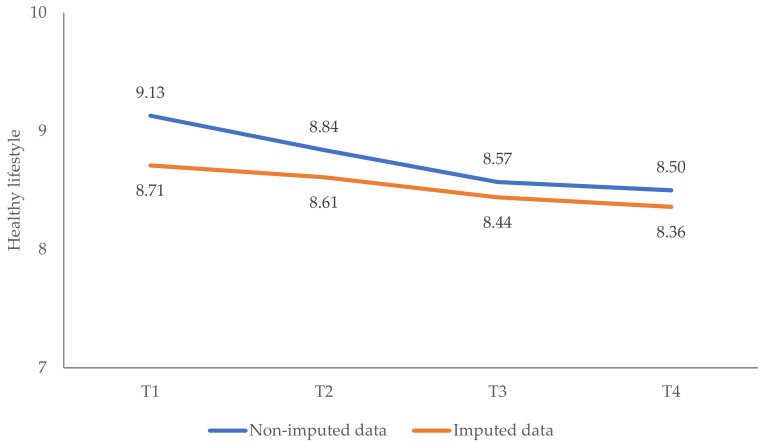
Changes over time in the subscale “Healthy lifestyle”: imputed vs. non-imputed results. Note: Imputed dataset: *n* = 1001. Non-imputed dataset: *n* = 218.

**Figure A6:**
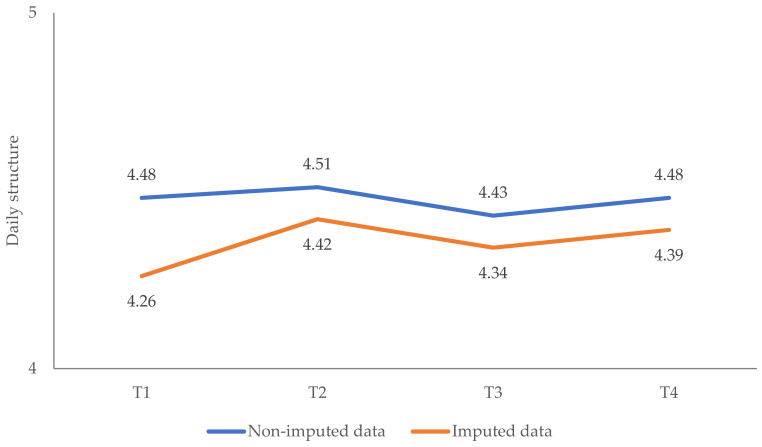
Changes over time in the subscale “Daily structure”: imputed vs. non-imputed results. Note: Imputed dataset: *n* = 1001. Non-imputed dataset: *n* = 218.

**Figure A7:**
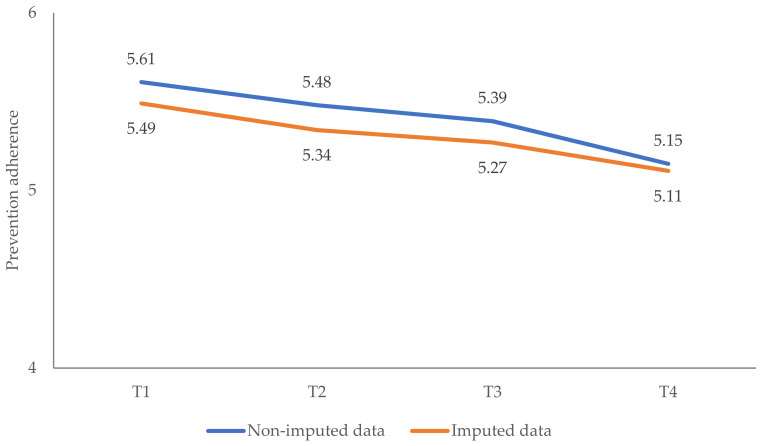
Changes over time in the subscale “Prevention adherence”: imputed vs. non-imputed results. Note: Imputed dataset: *n* = 1001. Non-imputed dataset: *n* = 218.

**Figure A8:**
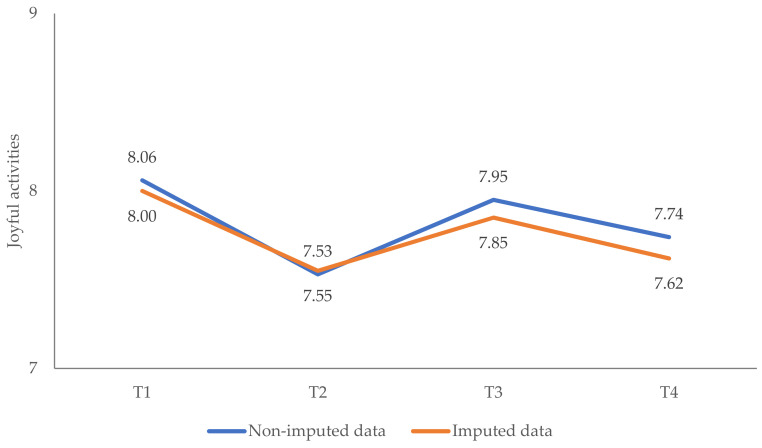
Changes over time in the subscale “Joyful activities”: imputed vs. non-imputed results. Note: Imputed dataset: *n* = 1001. Non-imputed dataset: *n* = 218.

## Data Availability

The entire dataset cannot be made publicly available since the analysis is still in progress. After the completion of the ongoing work, excerpts of the data can be obtained from the first authors upon reasonable request.

## Appendix A

### Appendix A.1. Multiple Imputation as Sensitivity Analysis

Complete case analysis was used for data analysis, in order to avoid additional layer of measurement error in the data [35]. To rule out a possible bias introduced by the exclusion of incomplete cases, we performed multiple imputation using Fully Conditional Specifications (FCS; [36,37]) as sensitivity analysis. FSC was chosen because this method is suitable for categorical as well as continuous predictors. The number of imputed data sets corresponded to the percentage of incomplete cases, namely: *m* = 80 for ADNM-8, PHQ-2, GAD-2, and pandemic-related coping strategies, and *m* = 90 for PC-PTSD-5. All variables of the respective data analysis were included in the imputation model. Additional variables, i.e., auxiliary variables, were selected for the imputation model based on their correlation with the incomplete variables in the present and earlier studies (e.g., [23]). Thus, an inclusive approach of multiple imputation was applied.

As illustrated in the charts below, the analysis with estimated missing values in the mental health outcomes (*n* = 1001) revealed comparable results. The mean scores of the mental health outcomes in the imputed dataset were slightly higher, but followed the same pattern as in the original, non-imputed dataset. Slightly higher values stemming from the imputed dataset might indicate that participants with less mental health problems were more likely to respond to all waves. With regard to pandemic-related coping behaviors, the scores stemming from the imputed data dataset were slightly lower, suggesting that participants who employed more coping behaviors were also more likely to respond to all waves. However, these assumptions would need to be tested in a more rigorous matter in order to draw reliable conclusions.
